# Supplementary material for: Modelling the structure of Short Gastrulation and generation of a toolkit for studying its function in Drosophila
Source: Biol Open. 2022 Jun 10;11(6):bio059199. doi: 10.1242/bio.059199 (PMC9194680; doi:10.1242/bio.059199)
Supplement: Supplementary information [file biolopen-11-059199-s1.pdf]

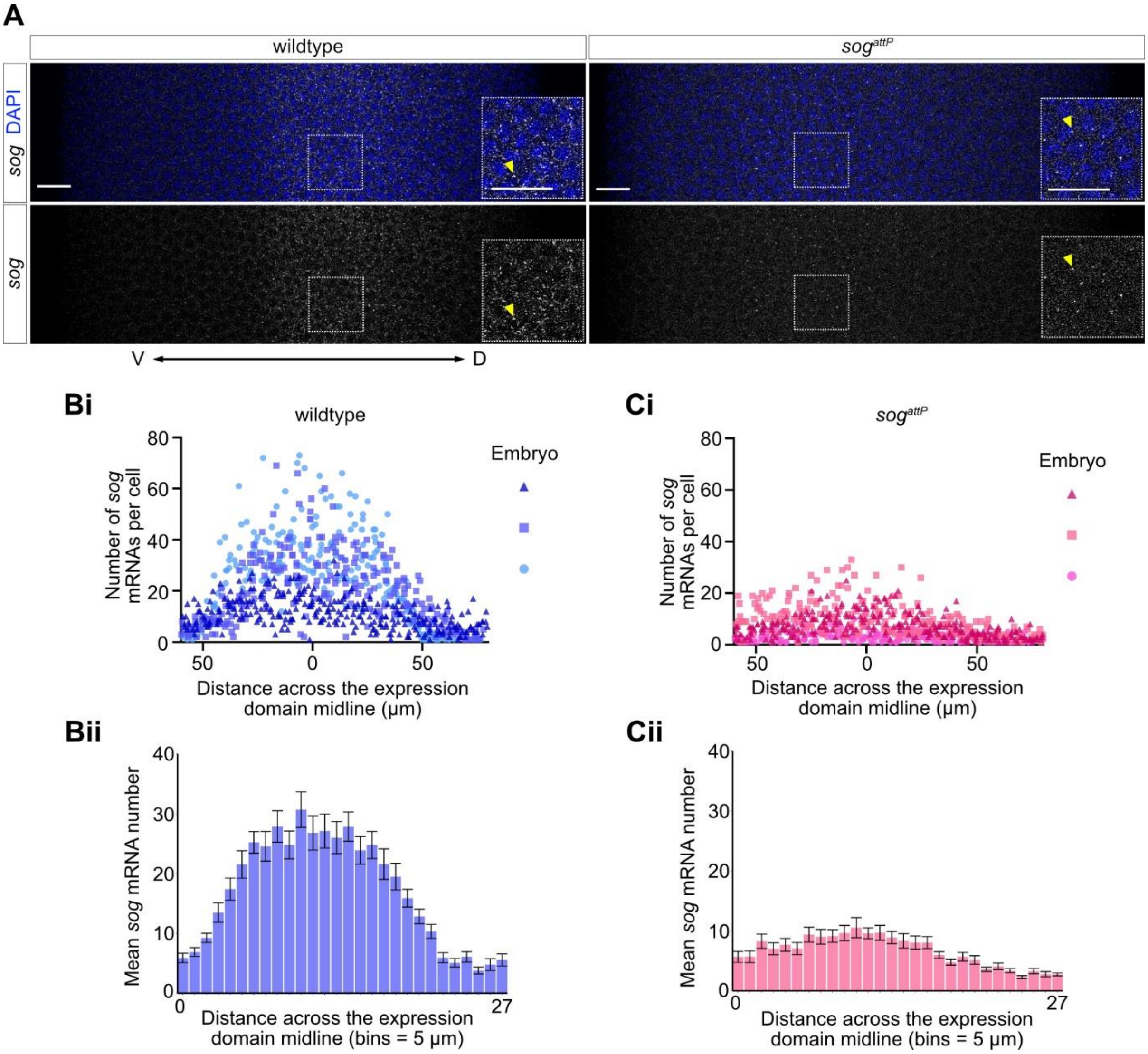

Fig. S1. *sog* is transcribed at low levels in early nc14 *sog*^{attP} embryos.

A) Confocal images (maximum intensity projections) showing *sog* mRNAs in early nc14
wildtype and *sog*^{attP} male embryos stained with *sog* smFISH probes and the DAPI nuclear marker. A merged image and single smFISH channel are shown for clarity. Spectrin antibody staining was used to measure membrane ingression to age the embryos (data not shown). Scale bar = 15 µm. Insets are enlarged sections of the *sog* expression domain (box), scale bar = 15 µm). Yellow arrowheads indicate transcription sites.

Bi) The numbers of *sog* transcripts per cell in three wildtype embryos were quantified and are plotted against distance from the *sog* expression domain midline (µm, - 60 = ventral edge). Data for each embryo is presented on the same graph in a shade of blue with a triangle, square, or circle symbol.

Bii) Data for each wildtype embryo were combined and binned according to distance across the *sog* expression domain (bins = 5 µm, each bin approximately corresponds to a nuclear

width, bin 0 = ventral edge). Mean *sog* mRNA number per bin is plotted against bin number. Error bars = mean ± SEM.

Ci) As for Bi, but for *sog* mRNA counts in three *sog*^{attP} embryos. Each embryo is represented by a shade of pink and either a triangle, square or circle symbol.

Cii) As for Bii, but for *sog*^{attP} embryos.

Biology Open • Supplementary information

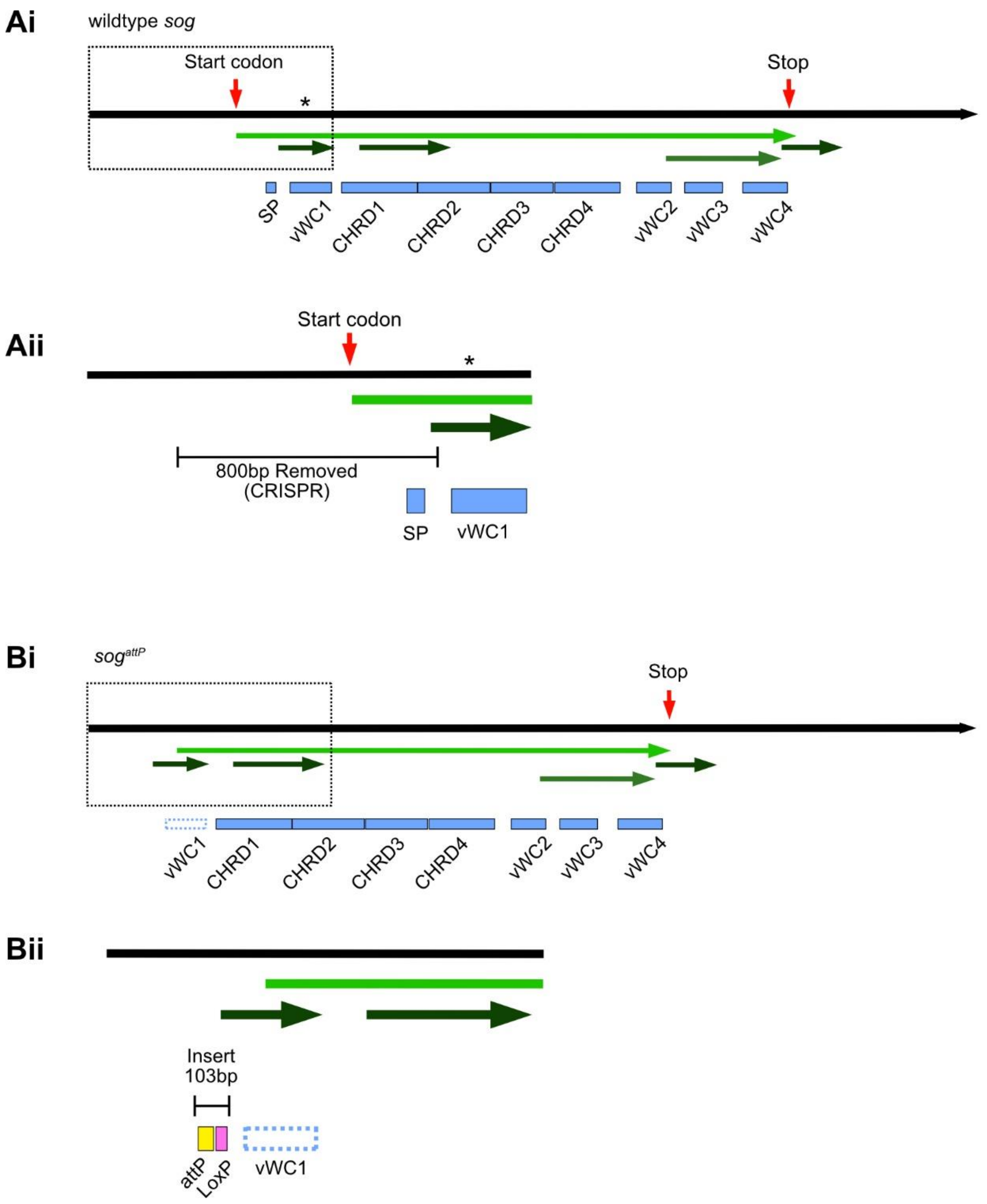

Fig. S2. Potential ORFs in the wild type *sog* and CRISPR modified *sog*^{attP} transcripts. Ai) Schematic showing the wildtype *sog* transcript (isoform E, (Larkin et al., 2021)). ORFs are shown by green arrows, with the shade of green indicative of the reading frame. A red arrow indicates the endogenous start and stop codons of the *sog* coding sequence. The asterisk marks the position where a truncated Sog ORF initiates following the CRISPR deletion (see Bi).

Biology Open • Supplementary information

Aii) An enlarged view of the outlined region in Ai) showing the 800 bp sequence removed by CRISPR-Cas9 and the locations of the predicted signal peptide (SP) and vWC1 domain (blue boxes).

Bi) Schematic showing the locations of predicted ORFs (green arrows) in the predicted *sog*^{attP} transcript (*sog* isoform E).

Bii) An enlarged view of the outlined region in Bi) showing the location of the 103 bp insert, containing attP and LoxP sequences (yellow and pink boxes, respectively), introduced into the sequence by CRISPR-Cas9 mediated homology directed repair, and the vWC1 domain. Due the CRISPR-Cas9 genome editing, none of the predicted ORFs contain the Sog SP, and therefore any translated protein would not enter the secretory pathway.

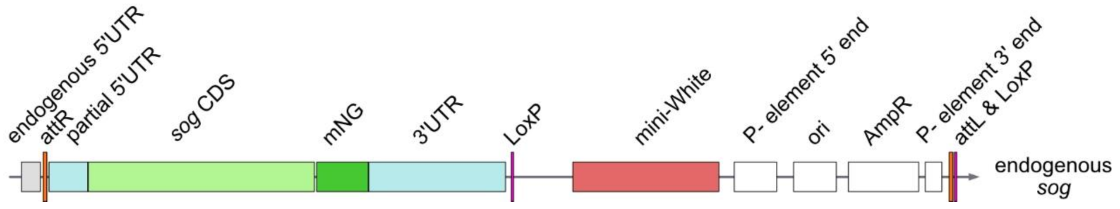

Fig. S3. The *sog* locus after CRISPR-Cas9 editing and reintegration of *sog-mNG* sequences.

The *sog* CDS and entire RIV white plasmid (Baena-Lopez et al., 2013) were inserted into the genome by phiC31 recombination, resulting in insertion of a 12,565 bp sequence. Due to the location of the CRISPR-Cas9 cut sites, some of the *sog* 5'UTR was removed in the initial CRISPR event. The grey box in the schematic represents the remaining endogenous 5'UTR, while the light blue box labelled 'partial 5'UTR' represents the 5'UTR sequence used to replace the 5'UTR sequences which were initially removed. The LoxP sequences (pink) located after the *sog* 3'UTR and P-element 3' end sequences permit removal of 6,081 bp after the 3'UTR by Cre-Lox recombination. However, this was not performed for this study.

Biology Open • Supplementary information

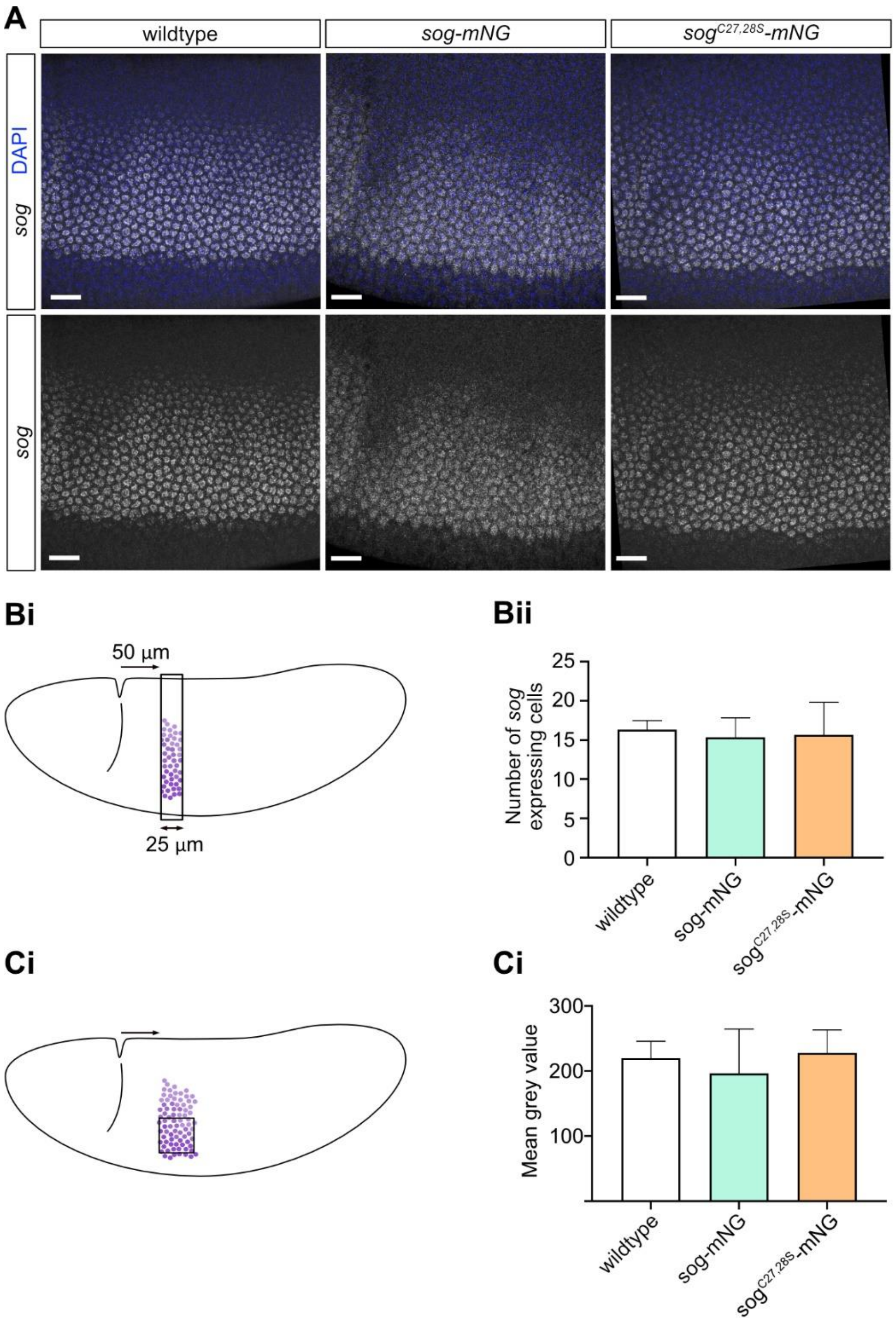

Fig. S4. *sog* expression in nc14 embryos is similar between genotypes.

A) *sog* mRNA was detected in wildtype, *sog-mNG*, and *sog*^{C27,28S}*-mNG* early stage 6 embryos by smiFISH. Scale bar = 20 µm.

Bi) Cartoon showing the position of a region of interest (ROI) that was used to measure the height of the expression domain based on the maximum number of *sog* expressing cells. The ROI is located 50 µm posterior of the cephalic furrow.

Bii) Graph shows the maximum number of *sog* expressing cells in the ROI depicted in Bi. No significant difference in the height of the *sog* expression domain was found (One-Way ANOVA

Biology Open • Supplementary information

with Tukey's multiple comparisons test, P>0.9). n = 3 embryos for each genotype, error bars show standard deviation.

Ci) Cartoon showing a 40x40 μm ROI, 50 μm to the posterior of the cephalic furrow, that was used to estimate *sog* expression level in early stage 6 embryos. Mean fluorescence intensity (based on grey value), of the ROI was measured, as clustering of *sog* mRNAs at this stage prevents the counting of absolute mRNA numbers as is possible in early nc14 embryos (see Fig S1).

Cii) Background subtracted fluorescence intensity (grey) values of the ROI are shown. No significant difference was detected between genotypes (One-Way ANOVA, Tukey's multiple comparisons test, P>0.7). n = 3 embryos for each genotype, error bars show standard deviation.

Biology Open • Supplementary information

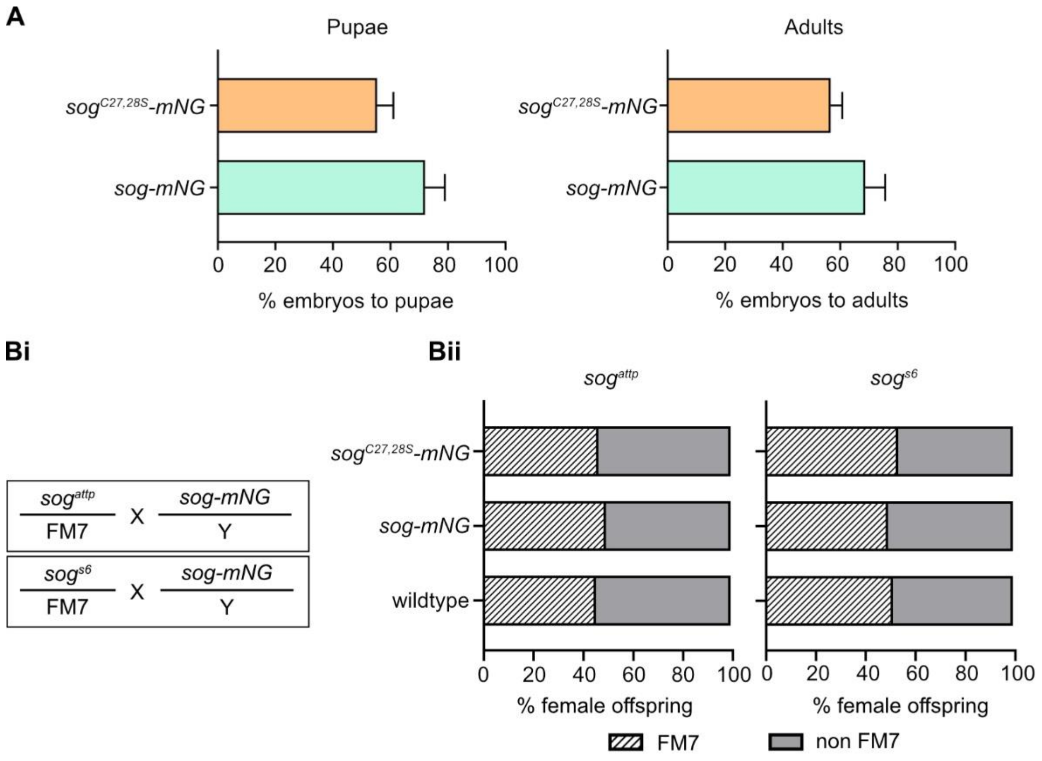

Fig. S5. Assessing viability of *sog-mNG* and *sog*^{C27,28S}*-mNG* flies.

A) Graphs show the percentage survival of *sog-mNG* and *sog*^{C28,28S}*-mNG* embryos to pupal and adult stages. No significant difference was found between the proportion of *sog-mNG* and *sog*^{C27,28S}*-mNG* flies that survived to pupae (p=0.14) and adulthood (p=0.21) (t-test, unpaired, two-tailed. t-tests were performed on raw data).

Bi) Overview of the crosses used to compare the ability of the *sog-mNG* and *sog*^{C27,28S}*-mNG* alleles to rescue either the strong *sog*^{S6} loss of function or *sog*^{attP} null alleles. Wildtype males were also tested as a control, only the *sog-mNG* allele is shown in the crossing scheme for simplicity. The number of female FM7 and non-FM7 progeny were counted. For female progeny that do not carry FM7, the *sog* allele has successfully rescued the *sog*^{attP} or *sog*^{s6} loss of function alleles.

Bii) The percentage of FM7 (striped bars) and non-FM7 (grey bars) progeny from the crosses described in Bi are shown. There is no significant association between the number of FM7/non-FM7 offspring and the genotype of fathers for crosses with *sog*^{attP} (X^2 (df = 2, N = 1764) = 2.07, p = 0.36) and *sog*^{S6} (X^2 (df = 2, N = 1596) = 1.71, p = 0.43) mothers. Statistical test was performed on raw data. Percentage frequency of the phenotypes is shown in the figure for simplicity.

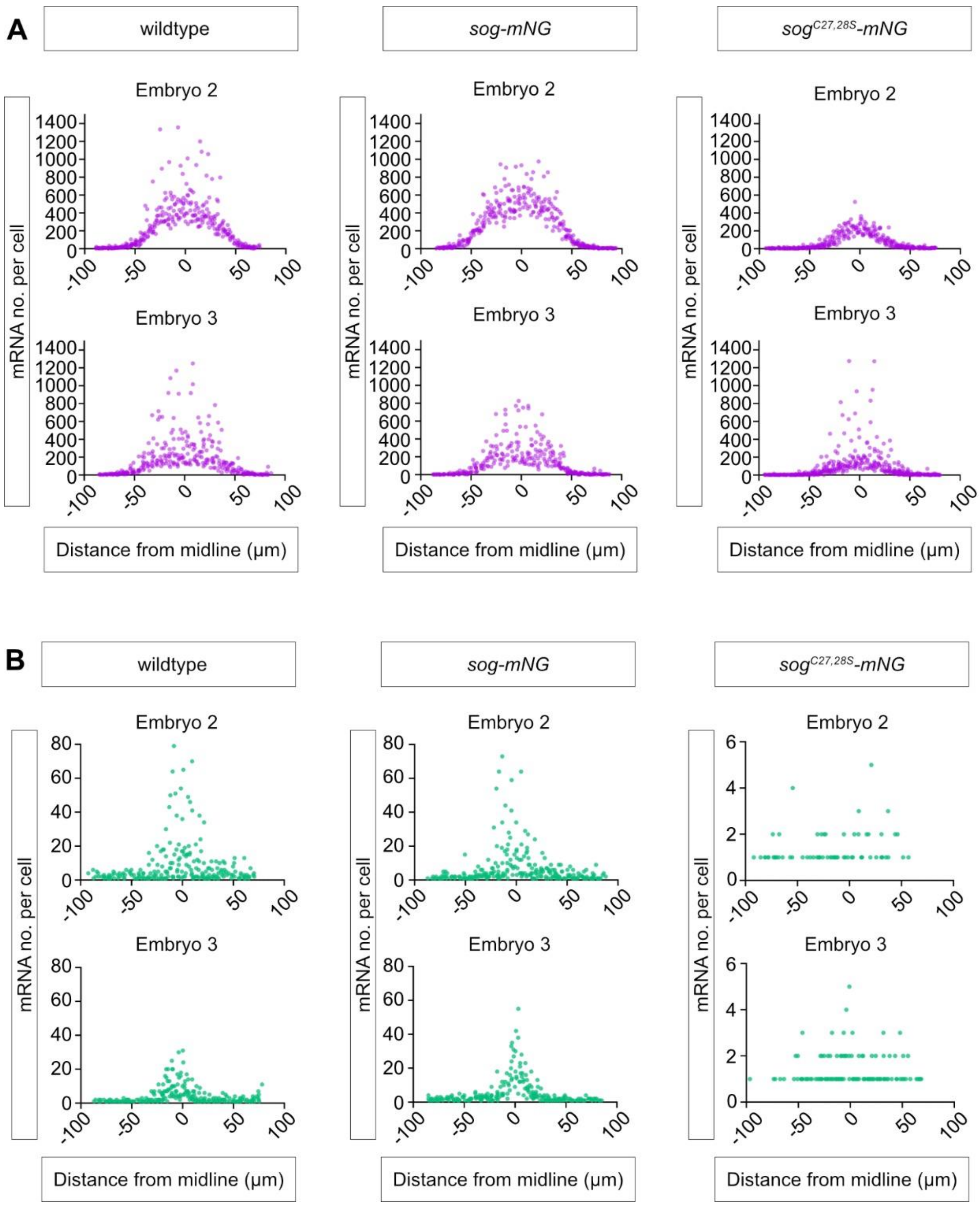

Fig. S6. Quantification of *ush* and *Race* expression.

(A) The numbers of *ush* transcripts per cell plotted against distance from the dorsal midline (µm, 0 = midline) for the additional biological repeats relating to Fig.4.

(B) As in (A), but for *Race* mRNAs.

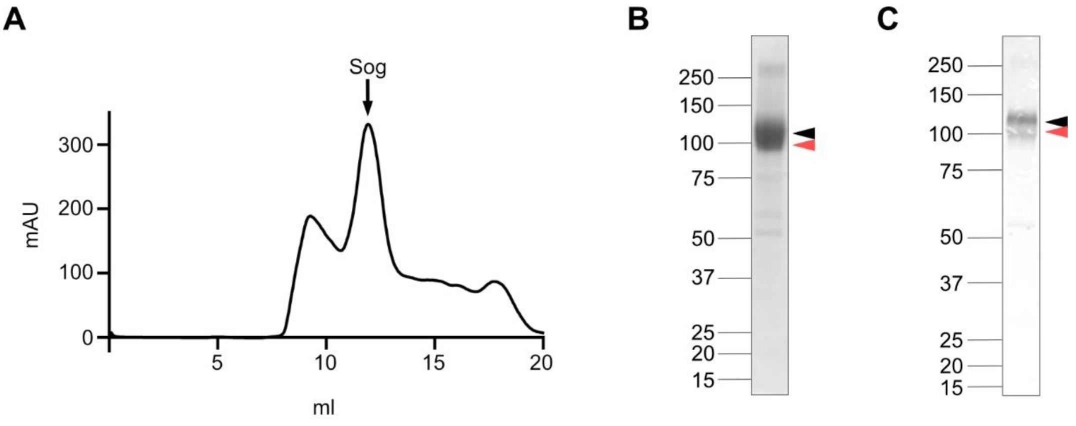

Fig. S7. Purification of Sog by size exclusion chromatography.

A) Following affinity chromatography using a His-tag, Sog was purified by SEC. The peak Sog-containing fraction, indicated by an arrow, was subjected to a second round of SEC (main text, Fig.6) prior to electron microscopy.

B) Reduced SDS-PAGE gel showing Sog from the peak fraction in A. The black arrow indicates full-length Sog and the red arrow indicates a Sog cleavage product.

C) Anti-His western blot of the same sample shown in B. Arrows are as in B.

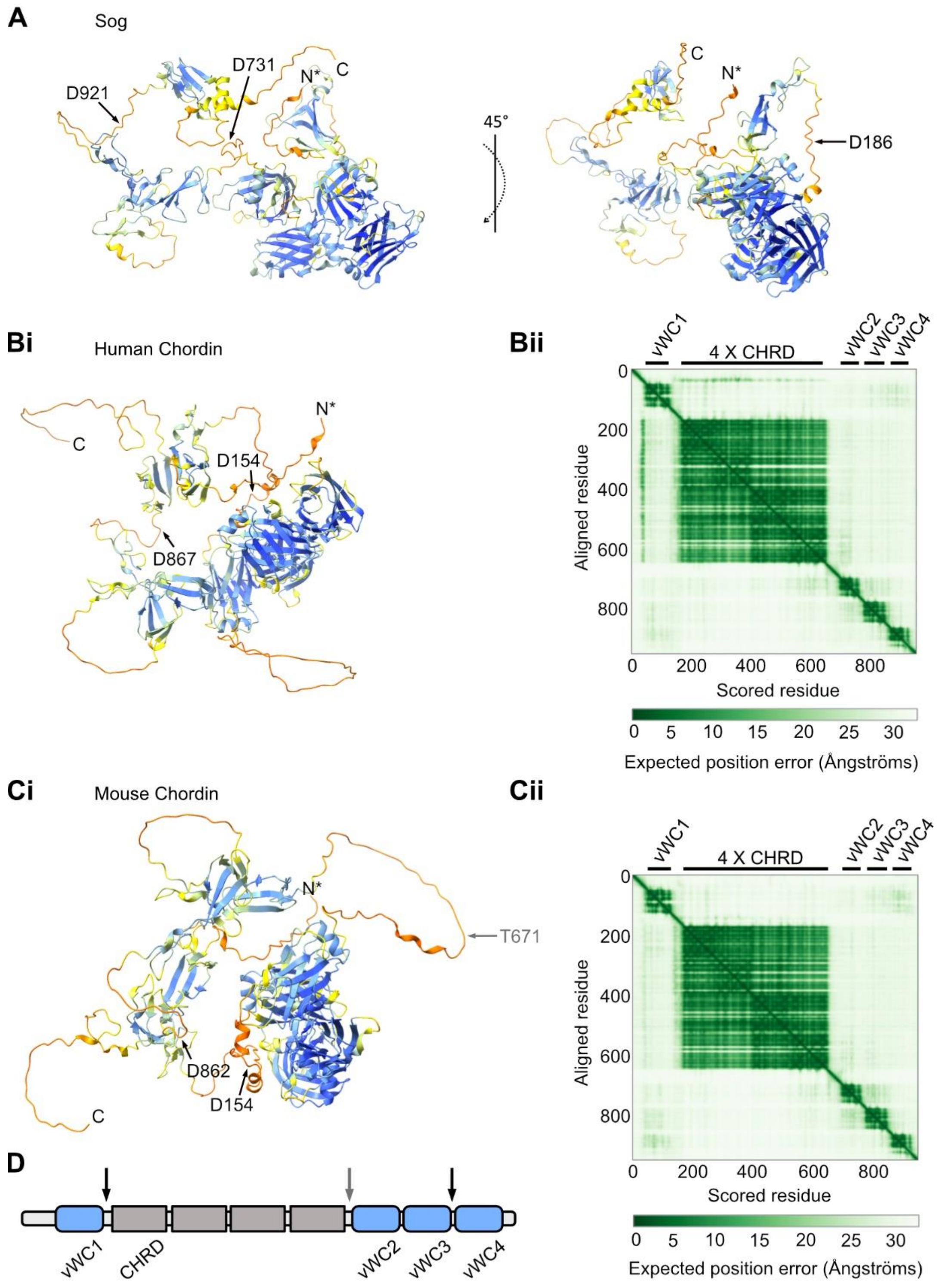

Fig. S8. Interdomain regions that contain the Sog/Chordin Tolloid cleavage sites are flexible.

A) AlphaFold2 structure prediction for Sog (Jumper et al., 2021; Varadi et al., 2022), colour coded according to per residue confidence (pLDDT) score (dark blue, very high confidence; light blue, confident; yellow, low confidence; orange, very low confidence). Tld cleaves Sog

Biology Open • Supplementary information

before D921, D731, and D186 (arrows) (Peluso et al., 2011; Shimmi & O'Connor, 2003) . In the figure shown, the Sog N-terminus is truncated at R79 (N*).

Bi) AlphaFold2 structure prediction for human Chordin (UniProt Q9H2X0), coloured as for Sog in (A). BMP1/Tld-like protease cleavage sites are annotated (arrows) (Scott et al., 1999). The predicted Chordin structure has been truncated following the signal peptide at G26 (N*).

Bii) Predicted aligned error (PAE) plot for the AlphaFold human Chordin prediction. Approximate boundaries for vWC and CHRD (4 X CHRD) domains are indicated by black bars above the heatmap.

Ci) AlphaFold2 structure prediction for mouse Chordin (Uniprot Q9Z0E2), coloured as for human Chordin and Sog. Tld cleavage sites are indicated by arrows. The predicted structure has been truncated after the signal peptide at G26 (N*). Two cleavage sites (D154 and D862) are conserved in human Chordin. A third Tld cleavage site (T671, grey arrow) in mouse Chordin is also used in the presence of Tsg *in vitro* (Scott et al., 2001).

Cii) Predicted aligned error (PAE) plot for the AlphaFold mouse Chordin prediction.

D) Cartoon of Chordin (not to scale), showing the vWC and CHRD domains in relation to Tld cleavage sites present in both mouse and human Chordin (black arrows), and the cryptic Tld target site (grey arrow) found in mouse Chordin in the presence of Tsg.

Biology Open • Supplementary information

References

Baena-Lopez, L. A., Alexandre, C., Mitchell, A., Pasakarnis, L., & Vincent, J.-P. (2013). Accelerated homologous recombination and subsequent genome modification in Drosophila. *Development*, *140*(23), 4818.

Jumper, J., Evans, R., Pritzel, A., Green, T., Figurnov, M., Ronneberger, O., Tunyasuvunakool, K., Bates, R., Žídek, A., Potapenko, A., Bridgland, A., Meyer, C., Kohl, S. A. A., Ballard, A. J., Cowie, A., Romera-Paredes, B., Nikolov, S., Jain, R., Adler, J., … Hassabis, D. (2021). Highly accurate protein structure prediction with AlphaFold. *Nature 2021 596:7873*, *596*(7873), 583–589.

Larkin, A., Marygold, S. J., Antonazzo, G., Attrill, H., dos Santos, G., Garapati, P. V, Goodman, J. L., Gramates, L. S., Millburn, G., Strelets, V. B., Tabone, C. J., Thurmond, J., Consortium, F., Perrimon, N., Gelbart, S. R., Agapite, J., Broll, K., Crosby, M., dos Santos, G., … Lovato, T. (2021). FlyBase: updates to the Drosophila melanogaster knowledge base. *Nucleic Acids Research*, *49*(D1), D899–D907.

Peluso, C. E., Umulis, D., Kim, Y.-J., O'Connor, M. B., & Serpe, M. (2011). Shaping BMP Morphogen Gradients through Enzyme-Substrate Interactions. *Developmental Cell*, *21*(2), 375–383.

Scott, I. C., Blitz, I. L., Pappano, W. N., Imamura, Y., Clark, T. G., Steiglitz, B. M., Thomas, C. L., Maas, S. A., Takahara, K., Cho, K. W. Y., & Greenspan, D. S. (1999). Mammalian BMP-1/Tolloid-Related Metalloproteinases, Including Novel Family Member Mammalian Tolloid-Like 2, Have Differential Enzymatic Activities and Distributions of Expression Relevant to Patterning and Skeletogenesis. *Developmental Biology*, *213*(2), 283–300.

Scott, I. C., Blitz, I. L., Pappano, W. N., Maas, S. A., Cho, K. W. Y., & Greenspan, D. S. (2001). Homologues of Twisted gastrulation are extracellular cofactors in antagonism of BMP signalling. *Nature*, *410*(6827), 475–478.

Shimmi, O., & O'Connor, M. B. (2003). Physical properties of Tld, Sog, Tsg and Dpp protein interactions are predicted to help create a sharp boundary in Bmp signals during dorsoventral patterning of the Drosophila embryo. *Development (Cambridge, England)*, *130*(19), 4673–4682.

Varadi, M., Anyango, S., Deshpande, M., Nair, S., Natassia, C., Yordanova, G., Yuan, D., Stroe, O., Wood, G., Laydon, A., Zídek, A., Green, T., Tunyasuvunakool, K., Petersen, S., Jumper, J., Clancy, E., Green, R., Vora, A., Lutfi, M., … Velankar, S. (2022). AlphaFold Protein Structure Database: Massively expanding the structural coverage of protein-sequence space with high-accuracy models. *Nucleic Acids Research*, *50*(D1).

Table. S1. Primer and oligonucleotide sequences.

Plasmid	Primer	Sequence (5'-3')
pU6-BbsI-chiRNA-guides	Guide 1 Forwards	[Phos]CTTCGAGTCGATCTCGTATGAGGA
	Guide 1 Reverse	[Phos]AAACTCCTCATACGAGATCGACTC
	Guide 2 Forwards	[Phos]CTTCGCATGCGCCGCTCATGTTCG
	Guide 2 Reverse	[Phos]AAACCGAACATGAGCGGCGCATGC
pTV-Cherry-homology-arms	Homolog arm 1 Forwards	CTAGCACATATGCAGGTACCTTTAAGATTGTCAGCATTGCA
	Homolog arm 1 Reverse	AGTTGGGGCACTACGGTACCATTACGACAACGCGACTTTT
	Homology arm 2 Forwards	CGAAGTTATCACTAGTAGTCCGACACGGGCAGGC
	Homology arm 2 Reverse	GGAGATCTTTACTAGTGCAACTCGGGAACATAATAG
pAC-p5UTR-Sog CDS-link	KpnI p5UTR F	GGTACCTCATACGAGATCGACTCTATTTTCC
	NotI SogCDS R	GCGGCCGCGCTGGAGGATCGCTGCT
	Linker sense	[Phos}GGCCGCCGGTGGTGGTGGAAGTGGCGGAGGTGGAGGGCC
	Linker antisense	[Phos]CTCCACCTCCGCCACTTCCACCACCACCGGC
pAC-mNeonGreen-sog3UTR	Kpn1 NG F	GGTACCATGGTGAGCAAGGGCG
	NotI NG R	GCGGCCGCTTACTTGTACAGCTCGTCCATG
	pACng3UTRinfFwd	CAAGTAAGCGGCCGCGCGGCTCCACGTGACGGAT
	pAC3UTRinfRev0818	ACCTTCGAAGGGCCCATGGGTATATTTCGAATATATTTTGTCTATATTTC
pAC-p5UTR-sogCDS-link-mNeonGreen-3UTR	ApaI NG F	GGGCCCATGGTGAGCAAGGGCG
	BstBI 3UTR R	TTCGAAATGGGTATATTTCGAATATATTTTGTCTATATTTCAATTTA
RIV-p5UTR-sog-link-	RIV p5UTR inffwd 250418	GGCGCGTACTCCACGAATTCTCATACGAGATCGACTCTAT
	Linker rev20818	TCCACCTCCGCCACT

Biology Open • Supplementary information

mNeonGreen-3UTR	Link-NGinfFwd0818	GTGGAAGTGGCGGAGGTGGAATGGTGAGCAAGGGC
	RIV 3UTR infrev 250418	GCGGCCGCTCCGGAGAATTCATGGGTATATTTCGAATATA
RIV-p5UTR-sogPALM-link-mNeonGreen-3UTR	RIV p5UTR inffwd 250418	GGCGCGTACTCCACGAATTCTCATACGAGATC GACTCTAT
	palm mut rev 161018	GCGGCGTCCTCGCTGTGAGAAGAGCTCCTTTCCAGGAGC
	palm mutfwd3 1218	TCTCACAGCGAGGACGCCGC
	RIV 3UTR infrev 250418	GCGGCCGCTCCGGAGAATTCATGGGTATATTTCGAATATA
**Probe**	**Primer**	**Sequence (5'-3')**
mNeonGreen-biotin-UTP	T3 promoter, forward.	ATTAACCCTCACTAAAGGGAATGGTGAGCAAGGGCGAGGAG GAT
	T7 promoter, reverse	GAATTAATACGACTCACTATAGGGATTACTTGTACAGCTCG TCCATGCC

Table. S2. Sequences of the *Race* smiFISH, *lacZ* Stellaris, and *sog* Stellaris and exonic smiFISH probes. *Race* smiFISH probes fused to Quasar 570-conjugated Y-FLAP, sog exonic smiFISH probes fused to Quasar 570-conjugated Z-FLAP, and *sog*, *ush* (Quasar 570 conjugated) and *lacZ* (Quasar 670 conjugated) Stellaris probes were used for smFISH.

Probe name	No.	Probe 5'-3'
race Y FLAP	1	TTACACTCGGACCTCGTCGACATGCATTATCCGTTAATTGCCCTAA
race Y FLAP	2	TTACACTCGGACCTCGTCGACATGCATTCCCCAATTAAAGGCTACT
race Y FLAP	3	TTACACTCGGACCTCGTCGACATGCATTTCGCGAAACAGTTCGCCA
race Y FLAP	4	TTACACTCGGACCTCGTCGACATGCATTTCCGTGGTGTGTACTACA
race Y FLAP	5	TTACACTCGGACCTCGTCGACATGCATTCGACCATGTCCACCGAAA
race Y FLAP	6	TTACACTCGGACCTCGTCGACATGCATTACCACCGCAGAAGTGTTT
race Y FLAP	7	TTACACTCGGACCTCGTCGACATGCATTTCGCTTTTGGTACACAGT
race Y FLAP	8	TTACACTCGGACCTCGTCGACATGCATTCGATCGTAAGTGCAACCT
race Y FLAP	9	TTACACTCGGACCTCGTCGACATGCATTTGGTTGGTTCGATCTGTT
race Y FLAP	10	TTACACTCGGACCTCGTCGACATGCATTACTCTGGAAGTCACTTCA
race Y FLAP	11	TTACACTCGGACCTCGTCGACATGCATTCTCAAGTGATTGCCCACA

Biology Open • Supplementary information

race Y FLAP	12	TTACACTCGGACCTCGTCGACATGCATTGGATGACTCTGGGGTCAG
race Y FLAP	13	TTACACTCGGACCTCGTCGACATGCATTGGGCTAGCAGAAACAGTC
race Y FLAP	14	TTACACTCGGACCTCGTCGACATGCATTTACCGCCAAAGTAGCCAG
race Y FLAP	15	TTACACTCGGACCTCGTCGACATGCATTCTCCTTGACCAGCGCTTG
race Y FLAP	16	TTACACTCGGACCTCGTCGACATGCATTTACTCCTTGGCCTGTATC
race Y FLAP	17	TTACACTCGGACCTCGTCGACATGCATTCCTTGTTCAGATTCTCCA
race Y FLAP	18	TTACACTCGGACCTCGTCGACATGCATTGTTGGTTCGCTTGGCCAG
race Y FLAP	19	TTACACTCGGACCTCGTCGACATGCATTCAGGCAGCTTCGGTTTCC
race Y FLAP	20	TTACACTCGGACCTCGTCGACATGCATTTGATGTTGGAGCCATAGG
race Y FLAP	21	TTACACTCGGACCTCGTCGACATGCATTTTTCTTTTCGTTCTCGTC
race Y FLAP	22	TTACACTCGGACCTCGTCGACATGCATTCTCGGCGGATATCTCATT
race Y FLAP	23	TTACACTCGGACCTCGTCGACATGCATTTCCTTCATGAACTTGGCC
race Y FLAP	24	TTACACTCGGACCTCGTCGACATGCATTCTTGGTGGTATCACTGGC
race Y FLAP	25	TTACACTCGGACCTCGTCGACATGCATTATTGGTACGAGCGCCATT
race Y FLAP	26	TTACACTCGGACCTCGTCGACATGCATTAACTGGCGCTTGAGATCC
race Y FLAP	27	TTACACTCGGACCTCGTCGACATGCATTAGCCCAGTTTGGTTAGAG
race Y FLAP	28	TTACACTCGGACCTCGTCGACATGCATTGTCTTCAGGTAGAGCAGC
race Y FLAP	29	TTACACTCGGACCTCGTCGACATGCATTTCCAGCAGTTCGGCATAG
race Y FLAP	30	TTACACTCGGACCTCGTCGACATGCATTACTCCATGGCGGAGAGTG
race Y FLAP	31	TTACACTCGGACCTCGTCGACATGCATTCCTTGACCTTGGCGAAAT
race Y FLAP	32	TTACACTCGGACCTCGTCGACATGCATTGCTATCCTTGTAGTCGCA
race Y FLAP	33	TTACACTCGGACCTCGTCGACATGCATTTCATCCAACCAGGCCTCG
race Y FLAP	34	TTACACTCGGACCTCGTCGACATGCATTCGAAGGTGTCGTCCTCGT
race Y FLAP	35	TTACACTCGGACCTCGTCGACATGCATTGATGTCCTCCAGCTGCTG
race Y FLAP	36	TTACACTCGGACCTCGTCGACATGCATTAGCGGACGAATATCCGCG
race Y FLAP	37	TTACACTCGGACCTCGTCGACATGCATTAGCCATGGATCTGCTGGT
race Y FLAP	38	TTACACTCGGACCTCGTCGACATGCATTCCTCAGGCGGAAACGCAC
race Y FLAP	39	TTACACTCGGACCTCGTCGACATGCATTACCGCGTCACCATAGTGT
race Y FLAP	40	TTACACTCGGACCTCGTCGACATGCATTTGGGTCCTGTCTCGGAGA
race Y FLAP	41	TTACACTCGGACCTCGTCGACATGCATTGCCCAATAGGTGCATGGG
race Y FLAP	42	TTACACTCGGACCTCGTCGACATGCATTCACTGCTGTGCCCACATG
race Y FLAP	43	TTACACTCGGACCTCGTCGACATGCATTCGATGTCCGCAATCTCTG
race Y FLAP	44	TTACACTCGGACCTCGTCGACATGCATTCTTCTCCGGAAAGGGGGA
race Y FLAP	45	TTACACTCGGACCTCGTCGACATGCATTAGCGCTCACATCCACCAG
race Y FLAP	46	TTACACTCGGACCTCGTCGACATGCATTTAGCCCTGCTTTTCCATC
race Y FLAP	47	TTACACTCGGACCTCGTCGACATGCATTGGAACATTTTGAGTGGCG
race Y FLAP	48	TTACACTCGGACCTCGTCGACATGCATTGAAGAAGTCGTCGCCCAT
sog_570	1	ATTCGATGGCGTTCGATTTC
sog_570	2	CAAGCAGACGATCAGCAGTC
sog_570	3	AATTCGCGCAAAACTTTGCC
sog_570	4	TACATAACTCCGAAGGGTGG

Biology Open • Supplementary information

sog_570	5	CCACACATTCACACTTGATG
sog_570	6	GGGCACTCGTTTTTGATATT
sog_570	7	GAGATGGGATCATCGCATTT
sog_570	8	TACATCCGTATCGTTTCGAT
sog_570	9	TAGCAACGCAGCGTAATGTT
sog_570	10	TGAGGAAATAGGAGGTGCGG
sog_570	11	TACATGGACTTCATTTCCTC
sog_570	12	CACATTCTGCGGATTGTAGG
sog_570	13	TTGTGGAACAGGAAACGGGC
sog_570	14	GCGATGAGGTGTAGAAGGAG
sog_570	15	AATTGAATGGCACGCGGACG
sog_570	16	GATAACACCCGCATCATCAA
sog_570	17	TGATAGACACTGAGAGTGCC
sog_570	18	GCAGAATGCGCTTGTAATCA
sog_570	19	GGAGGACAACATGGAGACGA
sog_570	20	AACTGAACAACTCCGTCTGC
sog_570	21	CATTGAAGACCAGGGTGAGA
sog_570	22	GCTCAATTTTCACACTCAGT
sog_570	23	CACACGTGGAATCTCATCGA
sog_570	24	GTATGGAAATGGGCGACGAC
sog_570	25	ACGCGACATCAGTCGAAGAT
sog_570	26	AGATGTGGGTACTTCTTGGA
sog_570	27	TCTGGAAGATTTCGCAGCTG
sog_570	28	ATCCATCGGTGTTCAAGTAG
sog_570	29	CAAACTGATGTTGGGCCTAT
sog_570	30	TGGTTGAAGTTGAAGCTCGG
sog_570	31	AACTTCTCCACACTACCAAT
sog_570	32	ATTGCACTCGTTGTCAATGG
sog_570	33	CGTTGAATTCCTCGAGCAAT
sog_570	34	AAGAAGCCTTCCAGATAGGA
sog_570	35	TGGAATGGACCTCCAGATAG
sog_570	36	AGCAGAAGCTGTTTGGAGTG
sog_570	37	AACATTGTTGTCCGTGTAGA
sog_570	38	CCGTTCACCAAATGGTTATC
sog_570	39	CGATTCGTTGTAGAAGCGTC
sog_570	40	CACATCTGACAGGAATCCTG
sog_570	41	CATTCACCATCACGCTGAAG
sog_570	42	GAGGATTGCGCTGAATAGTC
sog_570	43	CAGGAATGGATGCCAACTGG
sog_570	44	CTTCTTGTCTGGACGATAGG
sog_570	45	TCTTGTGTCCATTGGAACTG

Biology Open • Supplementary information

sog_570	46	CAGCACATTGGGATTGTTCG
sog_570	47	TTCTCGTACACCTTGTTGAC
sog_570	48	TGGGACATCAGGATCGGATG
sog Z FLAP	1	CCAGCTTCTAGCATCCATGCCCTATAAGATTGTAGGTGGTGTACATGG
sog Z FLAP	2	CCAGCTTCTAGCATCCATGCCCTATAAGTTGTGGAACAGGAAACGGGC
sog Z FLAP	3	CCAGCTTCTAGCATCCATGCCCTATAAGGCGATGAGGTGTAGAAGGAG
sog Z FLAP	4	CCAGCTTCTAGCATCCATGCCCTATAAGATAACACCCGCATCATCAAC
sog Z FLAP	5	CCAGCTTCTAGCATCCATGCCCTATAAGTGATAGACACTGAGAGTGCC
sog Z FLAP	6	CCAGCTTCTAGCATCCATGCCCTATAAGGCAGAATGCGCTTGTAATCA
sog Z FLAP	7	CCAGCTTCTAGCATCCATGCCCTATAAGAACTGAACAACTCCGTCTGC
sog Z FLAP	8	CCAGCTTCTAGCATCCATGCCCTATAAGCATTGAAGACCAGGGTGAGA
sog Z FLAP	9	CCAGCTTCTAGCATCCATGCCCTATAAGGCTCAATTTTCACACTCAGT
sog Z FLAP	10	CCAGCTTCTAGCATCCATGCCCTATAAGCACACGTGGAATCTCATCGA
sog Z FLAP	11	CCAGCTTCTAGCATCCATGCCCTATAAGACGCGACATCAGTCGAAGAT
sog Z FLAP	12	CCAGCTTCTAGCATCCATGCCCTATAAGATCCATCGGTGTTCAAGTAG
sog Z FLAP	13	CCAGCTTCTAGCATCCATGCCCTATAAGCAAACTGATGTTGGGCCTAT
sog Z FLAP	14	CCAGCTTCTAGCATCCATGCCCTATAAGTGGTTGAAGTTGAAGCTCGG
sog Z FLAP	15	CCAGCTTCTAGCATCCATGCCCTATAAGAACTTCTCCACACTACCAAT
sog Z FLAP	16	CCAGCTTCTAGCATCCATGCCCTATAAGATTGCACTCGTTGTCAATGG
sog Z FLAP	17	CCAGCTTCTAGCATCCATGCCCTATAAGCGTTGAATTCCTCGAGCAAT
sog Z FLAP	18	CCAGCTTCTAGCATCCATGCCCTATAAGAAGAAGCCTTCCAGATAGGA
sog Z FLAP	19	CCAGCTTCTAGCATCCATGCCCTATAAGTTGGAGTGCTTGGAATGGAC
sog Z FLAP	20	CCAGCTTCTAGCATCCATGCCCTATAAGCGATTCGTTGTAGAAGCGTC
sog Z FLAP	21	CCAGCTTCTAGCATCCATGCCCTATAAGCACATCTGACAGGAATCCTG
sog Z FLAP	22	CCAGCTTCTAGCATCCATGCCCTATAAGGAGGATTGCGCTGAATAGTC
sog Z FLAP	23	CCAGCTTCTAGCATCCATGCCCTATAAGCTTCTTGTCTGGACGATAGG
sog Z FLAP	24	CCAGCTTCTAGCATCCATGCCCTATAAGTCTTGTGTCCATTGGAACTG
lacZ 670	1	CTTCTTGGGGATTCCAATAG
lacZ 670	2	AATGGGATAGGTCACGTTGG
lacZ 670	3	GAACAAACGGCGGATTGACC
lacZ 670	4	TTGCACCACAGATGAAACGC
lacZ 670	5	TTCAGACGGCAAACGACTGT
lacZ 670	6	CGCGTAAAAATGCGCTCAGG
lacZ 670	7	TCCTGATCTTCCAGATAACT
lacZ 670	8	GAGACGTCACGGAAAATGCC
lacZ 670	9	ATCGCTGATTTGTGTAGTCG
lacZ 670	10	CACCCTGCCATAAAGAAACT
lacZ 670	11	CTCATCGATAATTTCACCGC
lacZ 670	12	ACGTTCAGACGTAGTGTGAC
lacZ 670	13	TAACGCCTCGAATCAGCAAC
lacZ 670	14	TGACCATGCAGAGGATGATG

Biology Open • Supplementary information

lacZ 670	15	CACGGCGTTAAAGTTGTTCT
lacZ 670	16	AGCGGATGGTTCGGATAATG
lacZ 670	17	GGGTTTCAATATTGGCTTCA
lacZ 670	18	GATCATCGGTCAGACGATTC
lacZ 670	19	GATCACACTCGGGTGATTAC
lacZ 670	20	GGATCGACAGATTTGATCCA
lacZ 670	21	CGCGTACATCGGGCAAATAA
lacZ 670	22	AAGCCATTTTTTGATGGACC
lacZ 670	23	TATTCGCAAAGGATCAGCGG
lacZ 670	24	GAAACCGCCAAGACTGTTAC
lacZ 670	25	CCTGTAAACGGGGATACTGA
lacZ 670	26	CATACAGAACTGGCGATCGT
lacZ 670	27	AAACTGCTGCTGGTGTTTTG
lacZ 670	28	CGCTATGACGGAACAGGTAT
lacZ 670	29	GTAGTTCAGGCAGTTCAATC
lacZ 670	30	ACCCAGCTCGATGCAAAAAT
lacZ 670	31	CTGACTGGCGGTTAAATTGC
lacZ 670	32	TTGTTTTTTATCGCCAATCC
lacZ 670	33	ACTTACGCCAATGTCGTTAT
lacZ 670	34	AATAAGGTTTTCCCCTGATG
lacZ 670	35	CAACGGTAATCGCCATTTGA
lacZ 670	36	GGAAGACGTACGGGGTATAC
lacZ 670	37	GTGGGCCATAATTCAATTCG
lacZ 670	38	TCAGTTGCTGTTGACTGTAG
lacZ 670	39	GGAAACCGTCGATATTCAGC
lacZ 670	40	CACCAGACCAACTGGTAATG
lacZ 670	41	GCCCGGTTATTATTATTTTT
lacZ 670	42	CTTACGCGAAATACGGGCAG
lacZ 670	43	TCCTTCACAAAGATCCTCTA
lacZ 670	44	TTGTCCAATTATGTCACACC
lacZ 670	45	AGTTCCATAGGTTGGAATCT
lacZ 670	46	CATTAAAGGCATTCCACCAC
lacZ 670	47	ATGGCATTTCTTCTGAGCAA
lacZ 670	48	CTCTTCTTTTTTGGAGGAGT

Biology Open • Supplementary information
